# LmABCB3, an atypical mitochondrial ABC transporter essential for *Leishmania major* virulence, acts in heme and cytosolic iron/sulfur clusters biogenesis

**DOI:** 10.1186/s13071-015-1284-5

**Published:** 2016-01-05

**Authors:** Marta Martínez-García, Jenny Campos-Salinas, María Cabello-Donayre, Estela Pineda-Molina, Francisco J. Gálvez, Lina M. Orrego, María P. Sánchez-Cañete, Sophie Malagarie-Cazenave, David M. Koeller, José M. Pérez-Victoria

**Affiliations:** Instituto de Parasitología y Biomedicina “López-Neyra” (IPBLN), CSIC, PTS Granada, Granada, Spain; Department of Molecular & Medical Genetics, Oregon Health & Science University, Portland, OR USA

**Keywords:** Heme trafficking and metabolism, Iron/sulfur clusters, Trypanosomatid parasites, Mitochondrial ABC transporter, *Leishmania*

## Abstract

**Background:**

Mitochondria play essential biological functions including the synthesis and trafficking of porphyrins and iron/sulfur clusters (ISC), processes that in mammals involve the mitochondrial ATP-Binding Cassette (ABC) transporters ABCB6 and ABCB7, respectively. The mitochondrion of pathogenic protozoan parasites such as *Leishmania* is a promising goal for new therapeutic approaches. *Leishmania *infects human macrophages producing the neglected tropical disease known as leishmaniasis. Like most trypanosomatid parasites, *Leishmania *is auxotrophous for heme and must acquire porphyrins from the host.

**Methods:**

LmABCB3, a new *Leishmania major* protein with significant sequence similarity to human ABCB6/ABCB7, was identified and characterized using bioinformatic tools. Fluorescent microscopy was used to determine its cellular localization, and its level of expression was modulated by molecular genetic techniques. Intracellular in vitro assays were used to demonstrate its role in amastigotes replication, and an in vivo mouse model was used to analyze its role in virulence. Functional characterization of LmABCB3 was carried out in *Leishmania* promastigotes and *Saccharomyces cerevisiae*. Structural analysis of LmABCB3 was performed using molecular modeling software.

**Results:**

LmABCB3 is an atypical ABC half-transporter that has a unique N-terminal extension not found in any other known ABC protein. This extension is required to target LmABCB3 to the mitochondrion and includes a potential metal-binding domain. We have shown that LmABCB3 interacts with porphyrins and is required for the mitochondrial synthesis of heme from a host precursor. We also present data supporting a role for LmABCB3 in the biogenesis of cytosolic ISC, essential cofactors for cell viability in all three kingdoms of life. LmABCB3 fully complemented the severe growth defect shown in yeast lacking ATM1, an orthologue of human ABCB7 involved in exporting from the mitochondria a gluthatione-containing compound required for the generation of cytosolic ISC. Indeed, docking analyzes performed with a LmABCB3 structural model using trypanothione, the main thiol in this parasite, as a ligand showed how both, LmABCB3 and yeast ATM1, contain a similar thiol-binding pocket. Additionally, we show solid evidence suggesting that *LmABCB3* is an essential gene as dominant negative inhibition of LmABCB3 is lethal for the parasite. Moreover, the abrogation of only one allele of the gene did not impede promastigote growth in axenic culture but prevented the replication of intracellular amastigotes and the virulence of the parasites in a mouse model of cutaneous leishmaniasis.

**Conclusions:**

Altogether our results present the previously undescribed LmABCB3 as an unusual mitochondrial ABC transporter essential for *Leishmania* survival through its role in the generation of heme and cytosolic ISC. Hence, LmABCB3 could represent a novel target to combat leishmaniasis.

**Electronic supplementary material:**

The online version of this article (doi:10.1186/s13071-015-1284-5) contains supplementary material, which is available to authorized users.

## Background

Leishmaniasis is a complex neglected tropical disease produced by different species of the protozoan parasite *Leishmania* [1]. This pathogen belongs to the Trypanosomatidae family, which also includes *Trypamosoma brucei*, the agent of sleeping sickness, and *Trypanosoma cruzi*, which is responsible for Chagas’ disease [2]. *Leishmania* has a digenetic life cycle that includes an extracellular flagellated promastigote form, which resides in the insect vector, and an intracellular amastigote form, which replicates inside the macrophages of the infected host and causes different forms of disease depending on the species [1]. According to the WHO and DNDi [3], leishmaniasis occurs in 98 countries with 350 million people living at risk. Each year, there are an estimated 1.3 million new cases, 30–40,000 deaths and 1,974,000 DALYs (the sum of years of potential life lost due to premature mortality and the years of productive life lost due to disability). Due to the lack of surveillance systems and the frequent occurrence of the disease in remote areas and marginalized populations [3]these are likely underestimates of its global impact. In the absence of vaccines, chemotherapy remains the main weapon to fight this disease. However, the limited efficacy and high toxicity of the drugs available, together with the emergence of numerous drug-resistant strains, have severely compromised their utility. Thus, there is an urgent need to find new treatments for this neglected disease.

Like most trypanosomatid parasites, *Leishmania* is auxotrophous for heme and must acquire this essential porphyrin from the host [4, 5]. Therefore, proteins involved in porphyrin uptake and intra-cellular trafficking could represent attractive targets for drug development. *In vitro*, *Leishmania* promastigotes can obtain heme through two independent routes [6, 7]. Hemoglobin-bound heme is taken up via receptor-mediated endocytosis [8, 9] and routed to the lysosome via early LRab5- and late LRab7-containing endosomes, followed by digestion of the globin protein [10, 11]. The trafficking of the released heme to the mitochondria requires the intracellular ATP Binding Cassette (ABC) transporter LABCG5 [7]. *Leishmania* promastigotes can also obtain heme from an exogenous supplement of free hemin [6] by an active transport process [7] which depends on a specific heme transporter called LHR1, recently identified in *L. amazonensis* [12]. LHR1 is essential for viability of the parasite [12] and the deletion of even one *lhr1* allele severely reduce virulence in an *in vivo* model of cutaneous leishmaniasis [13], validating LHR1 as a promising drug target. Regardless of the mechanism of import, heme must get to the mitochondria, where it is required for the synthesis of the heme-containing cytochromes of the respiratory chain. To date the mechanism via which the parasite imports heme into mitochondria is unknown. In the case of intracellular amastigotes, it has been suggested that they could use coproporphyrinogen, a heme precursor from the macrophage, which could then be converted to heme in the mitochondrion of the parasite [14, 15]. Subsequently, the just synthesized heme would need to be exported to the parasite cytosol, where it would exert an essential role, through a not yet known mechanism.

In mammals, the mitochondrial ABC half-transporter ABCB6 has been proposed to be responsible for mitochondrial porphyrin uptake required for *de novo* heme biosynthesis [16]. This transporter has also been found at the plasma membrane [17], the Golgi apparatus [18], and endo-lysosomal vesicles [19]. ABC half-transporters are composed of both a transmembrane domain (TMD) and a nucleotide binding domain (NBD), and function as dimers [20]. In the case of mammalian ABCB6, an additional N-terminal TMD (TMD0) with no sequence homology to any known protein is involved in its endo-lysosomal targeting [21]. Close homologues of mammalian ABCB6 include the mitochondrial ABC half transporters ABCB7 (also from mammals) and ATM1 from *Saccharomyces cerevisiae*, which all have significant sequence homology [20]. These last transporters, located in the inner mitochondrial membrane, are involved in the biogenesis of cytosolic iron/sulfur clusters (ISC) [22–24]. ISC are ancient cofactors found in many enzymes involved in electron transport, enzyme catalysis and regulation of gene expression, and are therefore essential for cell viability in all three kingdoms of life [25, 26], including trypanosomatid parasites [27]. These vital cofactors are formed in the mitochondria by the ISC machinery [25, 27]. It has been suggested that ISC assembled in mitochondria are coordinated with glutathione (GSH) to form a GSH-ISC complex that is exported from mitochondria by ABCB7/ATM1 [28, 29] although the nature of the sulfur- and GSH- containing compound transported by ABCB7/ATM1 is still not known with certainty [24]. Then, the cytosolic ISC assembly (CIA) machinery uses this mitochondrial export product for the assembly of cytosolic and nuclear iron-sulfur proteins [24, 28]. Both mitochondrial ISC and cytosolic CIA machineries are quite conserved in trypanosomatid parasites [27]. ABCB7 is essential in mice [30], and in humans, ABCB7 mutations cause X-linked sideroblastic anemia and ataxia (XLSA/A), a rare disorder characterized by an early-onset of non- or slowly progressive spinocerebellar ataxia and mild to moderate anemia [31]. In yeast, deletion of *ATM1* causes a drastic reduction of growth on rich medium, and complete growth failure on minimal medium [32].

The genome of *Leishmania major* contains a gene (*LmABCB3*) with sequence similarity to human *ABCB6/7* [33], and in this work we describe its functional characterization. We have found that this protein contains a unique N-terminal extension, not found in any other ABC transporter outside the genus *Leishmania*, which includes a potential metal binding site and is required for the proper mitochondrial localization of the protein. Functional assays in *Leishmania* and yeast showed that LmABCB3 is involved in the biogenesis of mitochondrial heme and cytosolic ISC. Finally, we demonstrate that LmABCB3 is an essential protein required at minimal levels to allow parasite virulence in a mouse model of leishmaniasis.

## Methods

### Chemical compounds

DAPI dihydrochloride, MES hydrate, Yeast synthetic drop-out medium supplements without uracil, Yeast synthetic drop-out medium supplements without tryptophan, lithium acetate dihydrate, phorbol 12-myristate 13-acetate (PMA), dibasic potassium phosphate, monopotassium phosphate, sodium chloride, DL-dithiothreitol (threo-1,4-dimercapto-2,3-butanediol), Triton X-100 (4-(1,1,3,3-tetramethylbutyl) phenyl-polyethylene glycol), Laemmli sample buffer, sodium hydrosulfite, PMSF (phenylmethylsulfonyl fluoride), D-(+)-glucose, D-galactose, glycerol, lactate acid, ethanol and hemin were obtained from Sigma. 5′-fluoroorotic acid (FOA) was from Zymo Research. PPIX-Na was from Frontier Scientific. MitoTracker Red and anti-GFP were from Molecular Probes (Invitrogen).

### Strains, culture conditions and cell transfection

Promastigote forms of wild-type *L. major* (MHOM/IL/80/Friedlin) were maintained *in vitro* at 28 °C in modified RPMI-1640 medium (Invitrogen, Carlsbad, CA) supplemented with 10 % heat-inactivated fetal bovine serum (hiFBS, Invitrogen), as described previously [34]. Promastigotes were transfected with the different constructs and selected for the corresponding resistance as described previously [35]. The yeast background W303 ΔATM1 (MATa,*ade*2,*can*1-100 *leu*2-3,112,*trp*1-1,*ura*3-1 *his*3-11,15 *atm*1:HIS3::ade4), W303 ΔATM1 + ATM1 (MATa,*ade*2,*can*1-100,*his*3-11,15,*leu*2-3,112,*trp*1-1,*ura*3-,*atm*1:HIS3::*ade*4 [pMW114(URA3)]) has been previously described [36]. Plasmids were transformed into the strain W303ΔATM1 + ATM1, using the lithium acetate method [37]. Transformants were selected on 2 % w/v glucose SC (−Trp) plates.

### DNA manipulation

*LmABCB3* (GeneDB- *L. major*, Accession Code LmjF32.3080) was isolated from genomic DNA of *L. major* by PCR using primers pairs (see all primers used in Additional file 1: Table S2) mg1-mg2 and mg1-mg3. PCR products were cloned respectively into the *Leishmania* expression vector pXG (Strain B1288) and pXG-/GFP+ (strain B2863) [38], kindly provided by Dr. Stephen M. Beverley (Washington University School of Medicine, USA) and sequenced. In the case of *LmABCB3_∆UNE*, DNA was amplified using primers pairs mg4-mg5. Site-directed mutagenesis to replace lysine 675 for methionine (K675/M) to give *LABCB3*^*K/M*^ was carried out with QuikChange XL Site-Directed Mutagenesis (Stratagene, La Jolla, CA) using primers pairs mg6-mg7. To express *LmABCB3* in yeast, first, *LmABCB3* was amplified (mg8-mg9), cloned in pENTR^TM^/SD/D-TOPO^R^ (Invitrogen) and sequenced. Then, *LmABCB3* was cloned by recombination into the yeast expression vector PDR299 (kindly provided by Dr. Olivier Cagnac (EEZ-CSIC, Spain)).

### Gene deletion constructs containing *hygromycin* B *phosphotransferase* (HYG)

Targeted gene replacement of the *L. major LmABCB3* gene was performed as described by FJ Perez-Victoria *et al*. [39] for the case of the *L. donovani LdMT* gene. Briefly, a targeting DNA fragment was constructed in which the *hyg* gene (conferring resistance to hygromycin B), preceded by 400 bp of the 5′-untranslated region of the *L. major dhfr-ts* gene, was flanked by *LmABCB3* upstream (primers pairs mg10-mg11) and downstream (mg12-mg13) regions (Fig. 3a). The different fragments were amplified by PCR from genomic DNA using the indicated primers, subcloned into pGEM-T vector (Promega), and assembled in this vector. Log phase *L. major* promastigotes were transfected with 5 μg of the linearized DNA targeting constructions, generated by *SphI* and *Not I* digestion, by using the Amaxa Nucleofector System (Lonza). Transfected parasites were selected with 50 μg/ml hygromicin B in semi-solid culture medium as described in [39].

### Fluorescence microscopy

For mitochondrial labelling, promastigotes were incubated with 50 nM of MitoTracker Red (Molecular Probes) for 30 min a 28 °C, as previously described [7]. Cells were washed in cold PBS and processed by microscopic observation. Images were acquired with confocal Leica SP5 microscopy and deconvolved using Huygens Professional from Scientific Volume Imaging (http://www.svi.nl).

### Gene expression analysis

Total RNA was prepared from control and *LmABCB3*^*+/−*^cells by using a total RNA isolation kit (Roche Biochemicals). cDNA was synthesized from 1 μg of total RNA using qSCRIPT^TM^cDNA Synthesis kit (Quanta Biosciences, Inc.) according to the manufacturer’s instructions). The cDNA obtained was amplified with primers mg14 and mg15 for *LmABCB3* and with primers mg16 and mg17 for *GADPH* (as internal control). Quantitative PCR was performed with iTaq Universal SYBR Green Supermix (BIORAD).

### In vitro infection of THP-1 macrophages

THP-1 macrophages (Sigma) infections with *L. major* were performed as described [40]. Briefly, THP-1 macrophages were infected at 35 °C with stationary-phase promastigotes forms of control and *LmABCB3*^*+/−*^*L. major* parasites at a ratio of 1:10 (macrophages:parasites). Excess parasites were removed after 24 h by washing and the macrophages were further incubated for the indicated times at 37 °C in a 5 % CO_2_ atmosphere. Cells were then fixed, stained with DAPI and observed in a wide-field Olympus IX81 fluorescence microscopy. Parasites were quantified using an analyze counter of the Image J software (http://rsb.info.nih.gov/ij/) as described [41].

### Analysis of in vivo infection

The analysis of the in vivo infection was performed as described in [34] with some modifications. Briefly, six-week-old male C57BL/6 J mice (Charles River Breeding Laboratories) were maintained in the Animal Facility Service of our Institute under pathogen-free conditions. Animals (seven mice/group) were injected subcutaneously (s.c.) in their left hind footpads with 10^6^ *L. major* stationary promastigotes resuspended in PBS (1.2 mM KH_2_PO_4_, 8.1 mM Na_2_HPO_4_, 130 mMNaCl and 2.6 mMKCl adjusted to pH 7), as described above. Disease progression was monitored by determining the inflammation thickness and the area of the lesion of the infected footpad using a Digimatic Caliper (Mitutoyo, Japan) and comparing these values with the uninfected contralateral control footpad.

### Analysis of *de novo* synthesized heme

Synthesis of heme from its precursor PPIX was performed as previously described [7] with some modifications. Briefly, parasites were incubated in culture medium supplemented with 10 % heme free FBS for 16 h with or without 0.5 uM PPIX at 28 °C. Then parasites were washed, lysed with freeze/thawing cycles with liquid nitrogen and intracellular heme was measured with the Hemin Assay Kit (Sigma) and normalized for the amount of protein. *De novo* synthesized heme was the difference between heme levels measured in parasites incubated in the presence and the absence of PPIX.

### Hemin-agarose pull-down assays

Hemin-agarose pull-down experiments were performed as described previously [7] but solubilizing the membrane proteins before the pulldown assay. Briefly, 2 μg of membrane proteins of parasites expressing LmABCB3-GFP, obtained as described [42], were solubilized with 1 % Triton X-100 during 3 h at 4 °C. After ultracentrifugation, solubilized proteins were diluted 10-fold with dilution buffer (100 mMKPi pH 7.4, 150 mMNaCl, 1 mM sodium hidrosulfite and 1 mM PMSF) to decrease the final detergent concentration to 0.1 %. Washed hemin-agarose aliquots were equilibrated with cold dilution buffer containing 0.1 % Triton X-100 and incubated with 30 μg solubilized protein during 30 min at 4 °C. Then, samples were washed four times with cold washing buffer (50 mMKPi pH 7.4, 150 mMNaCl, 0.1 % Triton X-100 and 1 mM DTT) and eluted with 20 μl of Laemmly sample buffer. LmABCB3-GFP was detected by western blotting using a polyclonal antibody against GFP. When indicated, different concentrations of free hemin or PPIX-Na were included during incubation of the solubilized proteins with the hemin-agarose.

### Effects of the heterologous expression of LmABCB3 on the growth of yeast lacking ScATM1

W303 ΔATM1 + ATM1 and W303 ΔATM1 + ATM1 + LmABCB3 cells were plated on 2 % (w/v) glucose minimal (SD) media (6.7 g/L yeast nitrogen base without amino acids, 1.87 g/L yeast synthetic drop-out supplements without tryptophan) containing or not 1 mg/ml FOA and incubated at 30 °C for three days. W303 ΔATM1, W303 ΔATM1 + ATM1 and W303 ΔATM1 + ABCB3 were diluted into rich (YP) media (20 g/L peptone, 10 g/L yeast extract, 3.8 g/L MES) supplemented with the indicated carbon sources: (2 % (w/v) glucose, 3 % (w/v) galactose, 2 % (w/v) lactate, 2 % (w/v) glycerol or 2 % (v/v) ethanol) to an *A*_600_ = 0.05. After one and two days of growth at 30 °C, the *A*_600_ was measured.

### Model building and docking analysis

A model for the 3D-structure of *L. major* ABCB3 (aa 283–875) was built using Phyre2 molecular modeling server (http://www.sbg.bio.ic.ac.uk/phyre2/html/page.cgi?id= index) based on *L. major* ABCB3 complete protein sequence. DALI server (http://ekhidna.biocenter.helsinki.fi/dali_server/) calculated the 3D-structural superimposition of yeast ATM1 structure (pdb code: 1 MHY, aa) and *L. major* ABCB3 model. Docking calculations were run into the Autodock 4.0 program. The predicted model for LmABCB3 (aa 283–875) was prepared for docking through the AutodockTools interface (http://autodock.scripps.edu/). The trypanothione coordinates were uploaded from the PDB structure 4ADW and used as a potential ligand. None of the residues in the protein were used as constrains in the docking simulation. The number of ligand orientations to the protein that are samples was set as default.

### Bioinformatic tools used

Sequences retrieval using Uniprot (http://www.uniprot.org/) allowed a search for homology patterns through the BLAST (http://blast.ncbi.nlm.nih.gov/Blast.cgi?PROGRAM=blastp&PAGE_TYPE=BlastSearch&LINK_LOC=blasthome) and CLUSTALW (https://npsa-prabi.ibcp.fr/cgi-bin/npsa_automat.pl?page=npsa_clustalw.html) software packages.

The identification of undescribed and potential new regions in the UNE sequence of LmABCB3 was achieved by the SMART domain recognition resource. The membrane bound topology recognition software MEMSAT3 (http://bioinf.cs.ucl.ac.uk/?id=756) was used through the interactive platform for secondary structure prediction analysis PSIPRED (http://bioinf.cs.ucl.ac.uk/psipred/). The identification of a mitochondrial targeting sequence was performed using Mitoprot software (http://ihg.gsf.de/ihg/mitoprot.html).

To assess the phylogenetic analysis of mitochondrial ABCB transporter sequences, protein sequences were first aligned using MAFFT (http://mafft.cbrc.jp/alignment/server/). The resulting multiple alignments were subjected to phylogenetic analysis using the Maximum Likelihood method of the MEGA 6 software [43]. Initial tree(s) for the heuristic search were obtained automatically by applying Neighbor-Join and BioNJ algorithms to a matrix of pairwise distances estimated using a JTT model, and then selecting the topology with superior log likelihood value.

### Statistical analysis

Experiments were performed three times in duplicate. All data are presented as mean and error represents S.E.M. Statistical significance was determined by Student’s *t*-test. Significance was considered as *p* < 0.05.

### Ethics statement

All experiments were performed according to the National/EU Guidelines for the Care and Use of Laboratory Animals in Research and the approval of the Ethics Committee of the Spanish National Research Council (CSIC, file JMPV.1.14/CEEA).

## Results

### LmABCB3 is an atypical ABC half-transporter with a unique N-terminal extension (UNE)

**Fig. 1:**
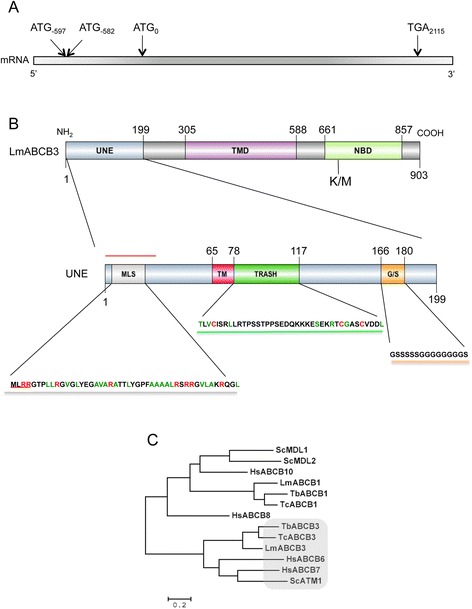
LmABCB3 contains a unique N-terminal extension (UNE). **a** Schematic representation of *LmABCB3* containing mRNA. In phase ATG codons found in 5‘ UTR and TGA Stop codon are indicated. **b** Schematic representation of LmABCB3 (*up*) showing the unique N-terminal extension (UNE), the Transmembrane Domain (TMD) and the Nucleotide Binding domain (NBD), indicating the K^675^M mutation (K/M) in the conserved Walker A motif that inactivates the protein. The schematic representation of the UNE region (*down*) highlights several motifs and sequences: i) a putative Mitochondrial Localization Signal (MLS) with conserved MLRR motif (*underlined in red*) and Arg (*red*) and hydrophobic Ala, Val and Leu residues (*green*); ii) a putative transmembrane segment (TM); iii) a TRASH domain with conserved Cys probably involved in metal co-ordination (*red*) and other residues (*green*) conserved in 70 % of TRASH domains [50]; iv) a Glycine/Serine repeat and iv) a putative signal peptide (*red line*). **c** Phylogenetic analysis of mitochondrial ABCB transporter sequences from *Leishmania*, mammals and yeast. Aligned protein sequences were subjected to phylogenetic analysis as described in Methods. The human representative of each mammalian subfamily was incorporated in the analysis to define each subfamily. Lm: *L. major*; Tb: *T. brucei*; Tc: *T. cruzi*; Hs: *H. sapiens*; Sc: *S. cereviciae*

Initially, we used human ABCB6, a known mitochondrial porphyrin transporter, as a template to identify putative homologs in the *L. major* genome database. This preliminary Blast analysis identified a potential *L. major* candidate, named ABCB3 (GeneDB-*Leishmania major*, Accession Code LmjF.32.3080) by Leprohon et al. [33]. This gene was annotated as a 2112 nucleotide gene coding for a 704 amino acids protein of 77.39 kDa. However, the *L. major* ABCB3 gene also contains two additional in phase ATG codons (ATG_−597_ and ATG_−582_ in Fig. 1a) [44] upstream of the annotated initial ATG (ATG_0_ in Fig. 1a). ATG_−582_is conserved in all *Leishmania* genomes analyzed whereas ATG_−597_ is found exclusively in *L. major*. Interestingly, none of these upstream ATG codons are present in the syntenic genes in the *T. cruzi* and *T. brucei* genomes (Additional file 2: Figure S1). Use of ATG_−597_ predicts a coding region of 2709 nt, and a protein of 903 amino acids with a molecular weight of 97.7 kDa, including a unique N-terminal extension (UNE) of 20 kDa (Fig. 1b). This UNE includes 199 amino acids with a predicted isoelectric point (Ip) of 9.5, in contrast to the rest of the protein, which is more acidic (Ip of 5.9). A blast analysis using the UNE region showed that it was found exclusively in *Leishmania* ABCB3, and that it was not present in any other sequenced genome, including those of the related organisms *T. brucei* and *T. cruzi* (Additional file 2: Figure S1). Similarly, there was no significant sequence similarity between the UNE region and the unique TMD0 domain from HsABCB6. Moreover, although secondary structure prediction analysis proposed a mainly helical configuration together with non-structured regions (possibly involved in protein-protein interactions) we have not been able to find a structural homologue for this extra sequence through different structural homology model based search programs [45]. However using both a domain identification resource (SMART, [46]) and a secondary structure prediction algorithm (Psipred, [47]) new potential sub-regions that may provide a lead to the function of this extension have been identified (Fig. 1b).

There are several motifs and sequence elements present in the UNE region, including a signal peptide sequence spanning from the N-terminal methionine to position 40 (Signal-BLAST, [48]). The sequence from amino acids 45 to 65 encodes a potential transmembrane segment as proposed by MEMSAT3 [49], although this result has not been confirmed by other available membrane topology prediction programs. A putative metal binding domain (aa 78–117) belonging to the TRASH super family was identified using the SMART algorithm. TRASH domains contain a well-conserved cysteine containing motif involved in metal coordination, represented in LmABCB3 by cysteines 78, 105, and 109 (Fig. 1b), and play roles in metal sensing, regulation and trafficking and in heavy-metal resistance [50]. Finally, a Glycine/Serine repeat (aa 166–180) was located next to the described metal-binding site. Similar amino acid repeats with diverse functions as structural components of proteoglycans [51] or as hinge sequences in inmunoglobulins [52] have been reported.

Finally, a comparative analysis between LmABCB3 and representative mitochondrial ABCB transporters using the CLUSTALW software [53, 54] (Additional file 3: Table S1) showed the highest degree of similarity with HsABCB6 (31.0 % identity), HsABCB7 (29.3 % identity) and ScATM1 (27.8 % identity) and the lowest (23.6–18.1 % identity) with HsABCB8, HsABCB10, ScMDL1 and ScMDL2. The sequence identity of LmABCB3 with the proteins encoded by the syntenic genes in *T. brucei* and *T. cruzi* was 45.7 % and 49.0 %, respectively. A phylogenetic analysis of this family of proteins indeed confirmed that LmABCB3 belonged to the HsABCB6/7-ScATM1 cluster (Fig. 1c).

### LmABCB3 is localized to the mitochondrion

**Fig. 2:**
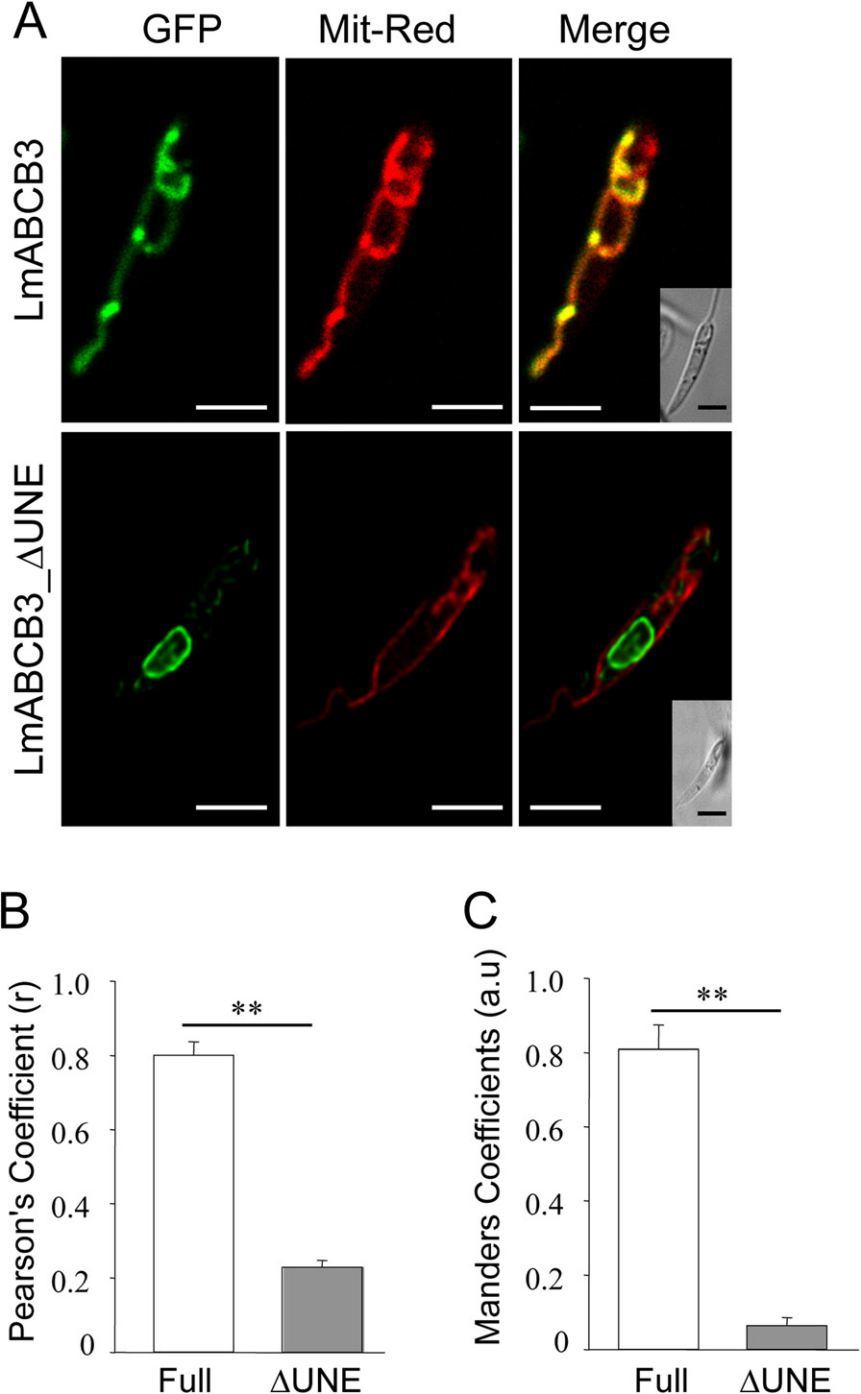
The UNE region is required for the mitochondrial localization of LmABCB3. **a** Subcellular localization of LmABCB3. Representative picture *of L. major* promastigotes expressing LmABCB3-GFP or LmABCB3_∆UNE-GFP (GFP) were incubated at 28 °C with 50 nM of Mitotracker Red (Mit-Red) for 30 min at 4 °C. Nomarsky images are shown in the inset. Scale bar: 1 μm. The figure shows a representative parasite of a total population of parasites with a similar fluorescence pattern. **b**-**c** Quantitative colocalization analysis. Pearson’s coefficient (**b**) was calculated for each individual deconvolved image. Mander’s coefficients (**c**) were calculated to define the M1 and M2 as the proportion of pixels in one channel (M1 = green, GFP) that overlap with some signal in other channel (M2 = red, Mit-Red). Both coefficients were assessed using “JACoP”, a colocalization plugin available in Fiji software. Full: LmABCB3-GFP; ∆UNE: LmABCB3_∆UNE-GFP. ** *p* < 0.001

A theoretical analysis performed with MitoProtII software [55] indicated a higher probability for a mitochondrial localization of LmABCB3 when the UNE region was included (94.9 % vs 56.4 %). In order to confirm this prediction we used confocal microscopy to evaluate the localization of C-terminal GFP tagged full-length LmABC3, and a truncated version lacking the UNE (LmABCB3_∆UNE). Western blot analysis using antibodies against GFP confirmed the expression of both proteins (Additional file 4: Figure S2). Next, we compared the cellular distribution of these GFP-tagged proteins with the mitochondrial marker MitoTracker Red, and calculated the co-localization coefficients (Pearson’s and Mander’s) after image deconvolution. LmABCB3 co-localized completely with the mitochondrial marker (Fig. 2a) with a Pearson’s co-localization index of 0.80 ± 0.03 (Fig. 2b). In contrast, LmABCB3_∆UNE was not localized to the mitochondrion (Fig. 2a and b, Pearson’s index of 0.23 ± 0.01). The exclusion of LmABCB3_∆UNE from mitochondria was evidenced by Mander’s index of 0.07 ± 0.02 compared with the Mander’s index for full LmABCB3 (0.81 ± 0.06) (Fig. 2c). All together these results indicate that the UNE region was required for the proper trafficking of LmABCB3 to the mitochondrion. Analysis of the sequence of the UNE predicts the presence of an MLRR sequence at its amino terminus that is rich in arginines and hydrophobic amino acids (alanine, valine and leucine) (Fig. 1b), which is characteristic of mitochondrial localization signals found in trypanosomatid protozoa [56, 57].

### LmABCB3 is an essential protein for *Leishmania*

LmABCB3 is constitutively expressed in *L. major* promastigotes [44]. This fact, together with the dimerization requirement of ABC half-transporters to become functional, allowed us to attempt the down-regulation of LmABCB3 activity through a dominant-negative approach, as we have described previously for LmABCG5 [7] and LmABCG2 [34]. To do so, we introduced a mutation at position 675 (K^675^M, K/M) which eliminates a conserved lysine inside the Walker A motif (Fig. 1b), known to be critical for ATP hydrolysis in ABC transporters [58]. Similar mutations, even if present only in one catalytic domain, inactivated all known ABC proteins [58]. Indeed, the overexpression of different ABC half-transporters with an equivalent K/M substitution produced dominant-negative inhibition in many wild-type half-transporters [7, 34, 59]. Subsequently, we transfected into *L. major* constructs containing either wild-type *LmABCB3* or mutated *LmABCB3*^*K/M*^. Transgenic parasites were easily recovered after hygromycin selection using the wild-type construct, as expected. In contrast, it was impossible to obtain parasites expressing mutant LmABCB3^K/M^ in spite of more than eight independent transfection assays performed. This result suggested not only that LmABCB3 function was essential for *L. major* promastigotes, but also that the low levels of functional wild-type (Wt) dimer expected from the dominant negative approach used were not sufficient to cover the requirements of the parasite.

### Heterozygous deletion of *LmABCB3* allele severely reduces *L. major* virulence

**Fig. 3:**
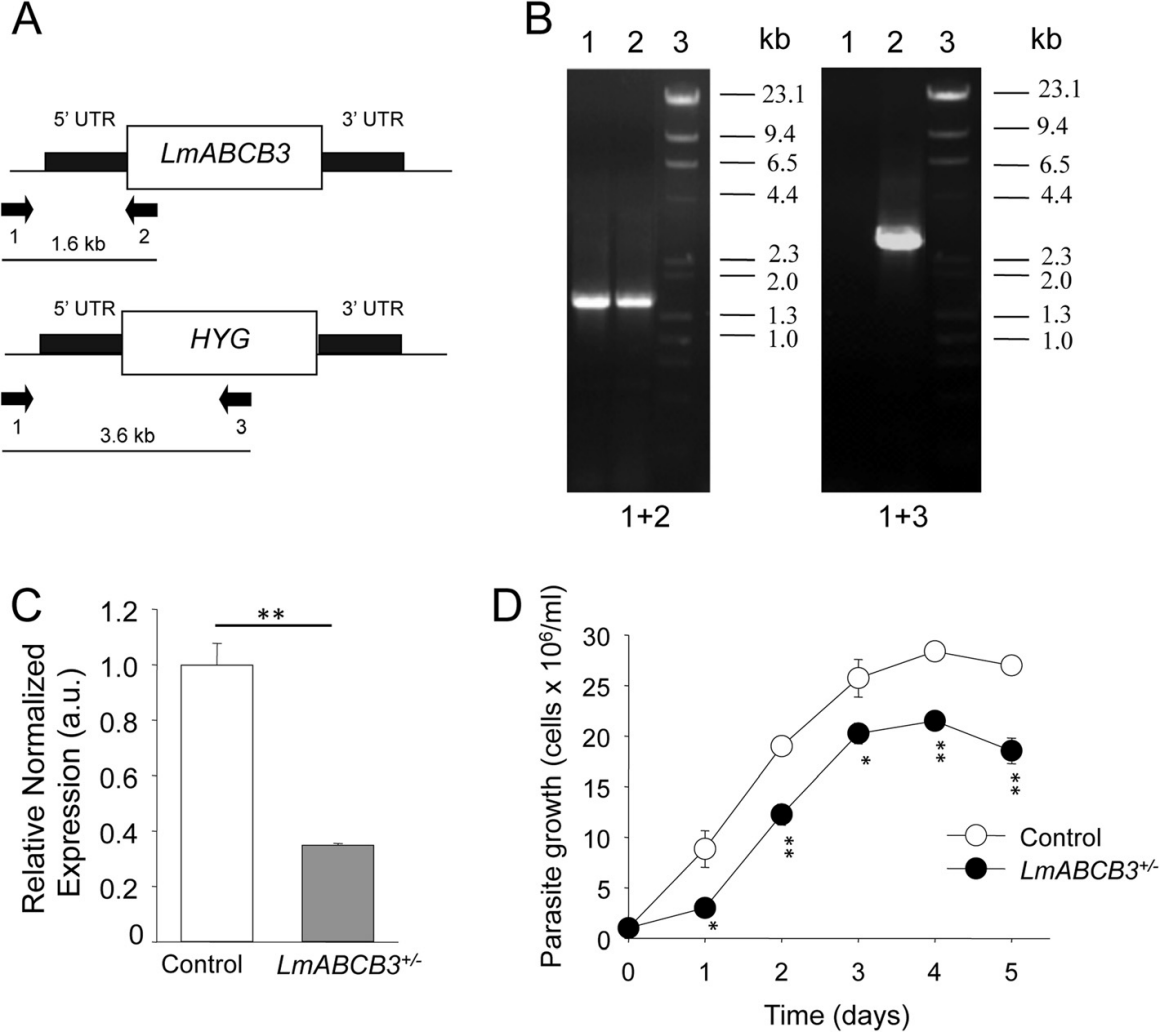
Generation and characterization of *L. major* promastigotes with one *LmABCB3* allele deleted. **a** Schematic representation of the *LmABCB3* locus and the hygromycin-resistance gene targeting construct used for gene replacement. The primers used (arrows 1–3) to verify the specific gene targeting and the expected sizes of the PCR-amplified products with the different pairs of primers are indicated. **b** PCR analysis of the *LmABCB3* locus in control and single knock out (*LmABCB3* ^*+/−*^) mutant promastigotes. The specific gene-targeting PCR product (primers 1 and 3, 3.6 kb) confirmed that replacement with the hygromycin-resistance gene in one LmABCB3 allele in *LmABCB3* ^*+/−*^parasites (+/−, lanes 2). Primers 1 and 2 amplified the expected 1.6 kb product in Wt (+/+, lanes 1) and in *LmABCB3* ^*+/−*^parasites. Lanes 3 shows the DNA marker used. **c** *LmABCB3* ^*+/−*^promastigotes have reduced *LmABCB3* expression. The expression level of *LmABCB3* from control and *LmABCB3* ^*+/−*^ *L. major* promastigotes was analyzed by qRT-PCR using mRNA isolated from each cell line as described in Methods. **p <0.001. **d** *LmABCB3* ^*+/−*^ parasites grow as axenic promastigotes. Growth curve obtained after cultivation of control (*white circles*) and *LmABCB3* ^*+/−*^(*black circles*) promastigotes during the indicated time. The results represent the mean ± SEM of three independent experiments. *p < 0.05; **p < 0.001

The dominant negative strategy produced important insights about an essential role of LmABCB3 but it did not allow us to perform further functional assays. We therefore deleted one allele of *LmABCB3* by homologous recombination following the strategy described in Fig. 3a, to obtain a heterozygous knock out line (*LmABCB3*^*+/−*^). Thus, parasites were transfected with a linearized hygromycin resistant cassette flanked by 5′ and 3′ *LmABCB3* UTR sequences. After hygromycin selection, a clone was recovered and its DNA isolated. The insertion of the hygromycin cassette in the right *locus* was confirmed by PCR (Fig. 3b): using a forward primer from the 5′ UTR region of the *LmABCB3* gene and a reverse primer from the hygromycin resistance gene, an amplification of the expected size was obtained only in *LmABCB3*^*+/−*^parasites. Analysis by real-time quantitative PCR (RT-qPCR) showed an approximate 60 % reduction in *LmABCB3 *mRNA level in *LmABCB3*^*+/−*^cells (Fig. 3c). Although gene expression in trypanosomatids is post-transcriptionally regulated, this result suggests a reduction in LmABCB3 protein level in *LmABCB3*^*+/−*^parasites, a point that could not be definitively confirmed due to the absence of specific antibodies. We could not obtain any double knock out line using a similar strategy with a second resistance cassette, in spite of many assays, consistent with the essential role of LmABCB3 as described above.

**Fig. 4:**
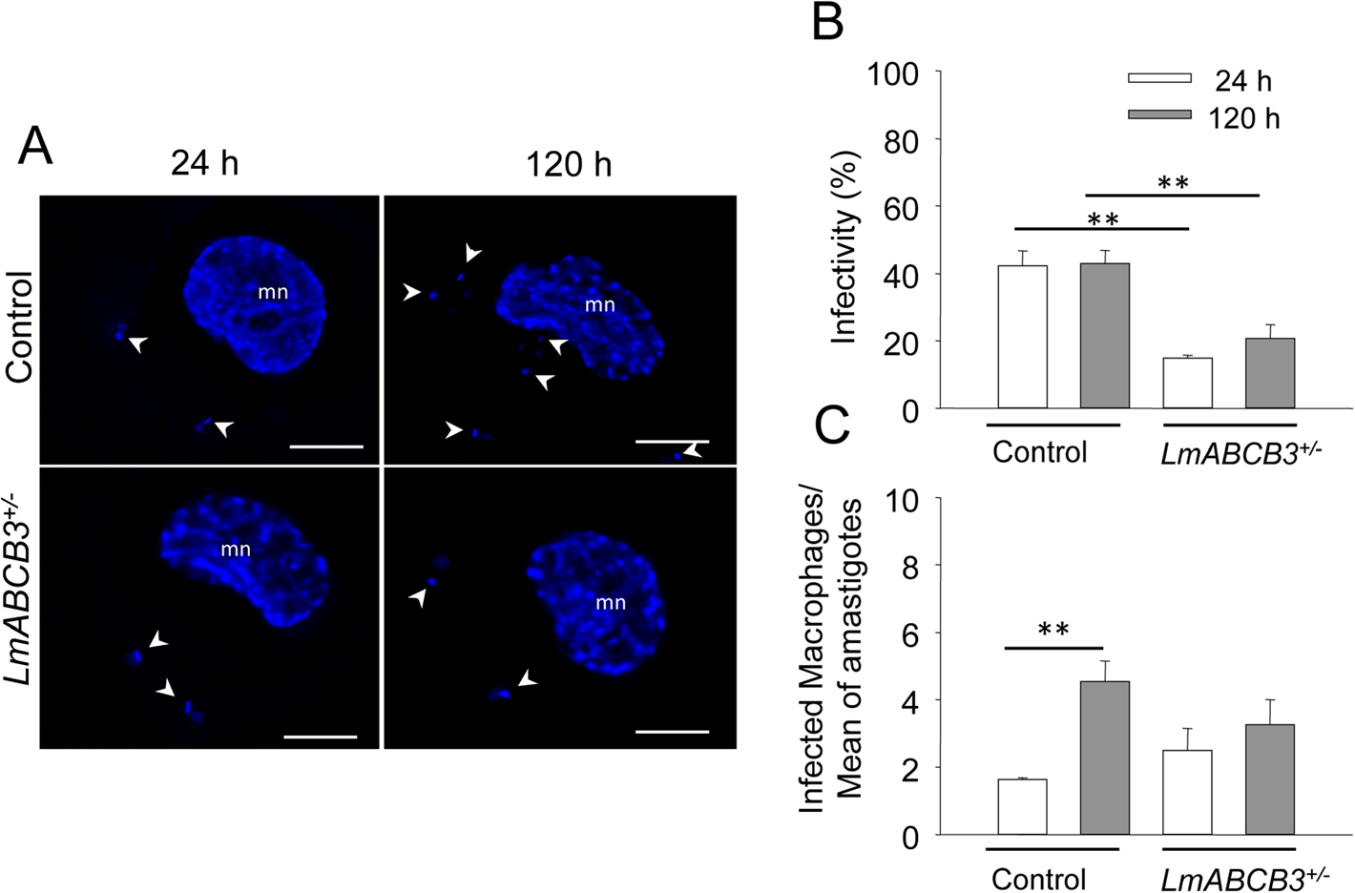
LmABCB3 is required for the intracellular replication of amastigotes. **a**. Infection of macrophages by control and *LmABCB3* ^*±/−*^ parasites. Representative picture of the infection of THP-1 macrophages with control and *LmABCB3* ^*+/−*^ stationary-phase *L. major* promastigotes, performed as described in Methods. At the indicated points, cells were fixed and DAPI (blue) stained. The macrophages nuclei (mn) and the kinetoplast and nuclei of intracellular amastigotes (arrowhead) are indicated. Scale bar: 5 um. **b** *LmABCB3* ^*+/−*^ parasites have reduced ability to infect macrophages. THP-1 macrophages were infected with control and *LmABCB3* ^*+/−*^promastigotes as above described and the percentage of infected macrophages (*n* = 300 macrophages/group) was calculated. The results shown are the means ± SEM of three independent experiments performed in duplicated. **p <0.0002. **c** *LmABCB3* ^*+/−*^parasites are unable to replicate as intracellular amastigotes. THP-1 macrophages were infected with control and *LmABCB3* ^*+/−*^ promastigotes as above described and the average number of intracellular amastigotes per infected macrophage was calculated at the indicated time points. The results shown are the means ± SEM. **p <0.0009

The reduced expression of LmABCB3in *LmABCB3*^*+/−*^cells resulted in a 30 % (*p* < 0.001) reduction in growth of promastigote parasites (Fig. 3d). This result contrasts with the dominant negative lethal effect above described, probably indicating that the level of functional LmABCB3 is higher in *LmABCB3*^*+/−*^ parasites than in dominant negative parasites. To compare the ability of control and *LmABCB3*^*+/−*^ parasites to infect host macrophages and replicate as intracellular amastigotes, THP-1 macrophages were infected with stationary-phase promastigotes from control and *LmABCB3*^*+/−*^ parasites. The percentage of infected macrophages and the number of intracellular amastigotes were quantified at 24 and 120 h post infection. Although *LmABCB3*^*+/−*^ parasites were capable of infecting macrophages (Fig. 4a), they did it at a significantly lower rate than control parasites (15 vs 42 %, *p* < 0.0002) (Fig. 4b). Significantly, during the 5 days post infection, the number of control amastigotes increased almost 3-fold (2.78-fold increase, *p* < 0.0009), whereas the *LmABCB3*^*+/−*^ parasites failed to complete a single replication cycle inside the macrophages (1.31-fold increase, *p* > 0.44) (Fig. 4c).

**Fig. 5:**
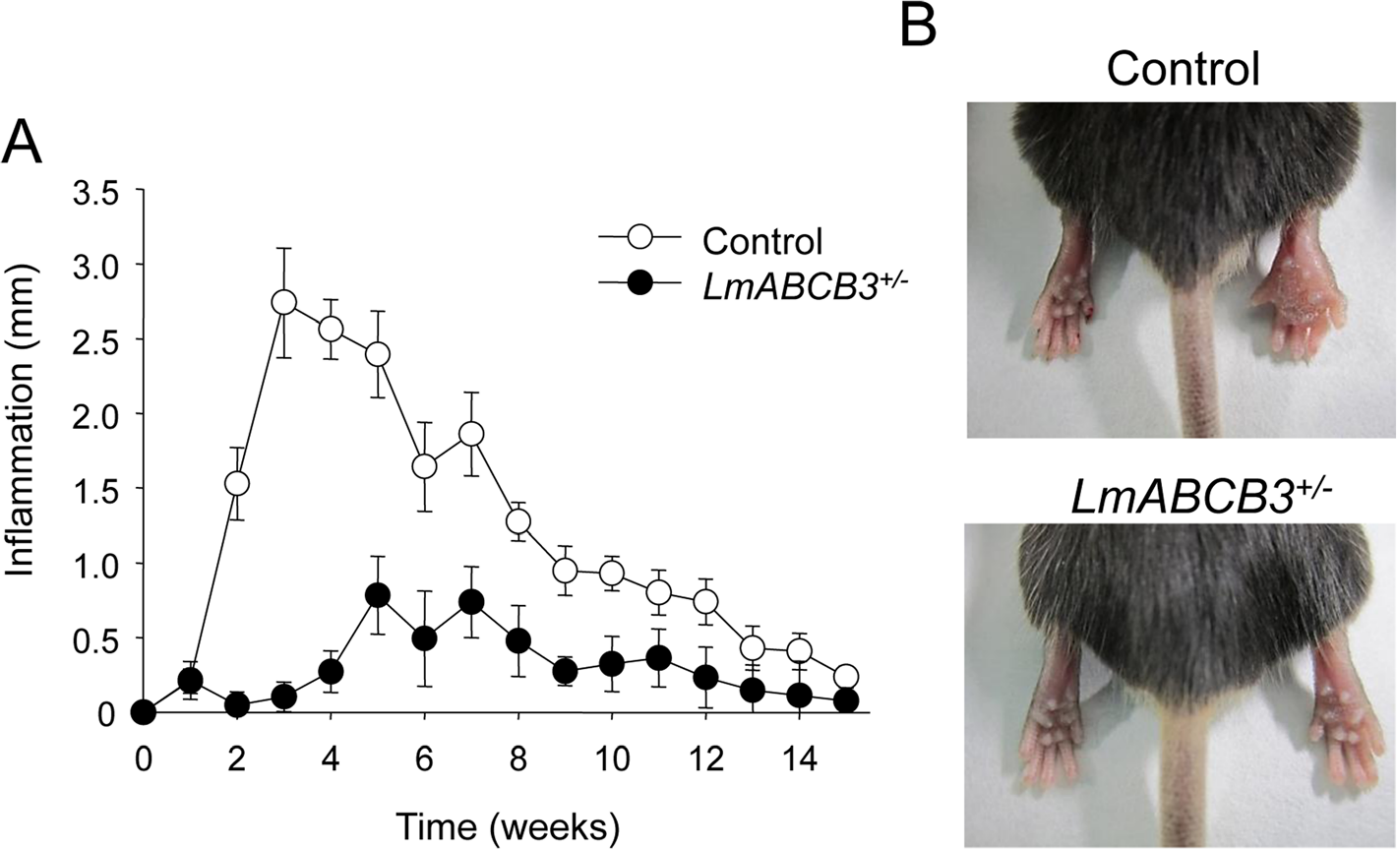
LmABCB3 is essential for *L. major* virulence. C57BL/6 male mice were infected with 1 × 10^6^ stationary-phase *L. major* promastigotes of control or *LmABCB3* ^*+/−*^ *L. major* in the left hind footpad. **a** Inflammation progression (difference between inoculated footpad and contralateral uninfected footpad) was recorded weekly. The values represent the means ± SEM of 7 mice. **b** Images show representative pictures of the footpad inflammation at 3 week post infection

As *LmABCB3*^*+/−*^ parasites did not replicate properly as intracellular amastigotes in the in vitro assay, we decided to analyze their ability to produce disease in an in vivo murine model of cutaneous leishmaniasis. C57BL/6 mice were infected with 10^6^ control or *LmABCB3*^*+/−*^ stationary-phase promastigotes by s.c. footpad inoculation, and inflammation in the footpad was monitored weekly for 15 weeks (Fig. 5). Mice infected with control parasites started developing progressive inflammation and edema around the injected site by the second week, reaching a maximum level at three weeks, after which the footpad began to recover slowly as expected for this model. In contrast, mice infected with *LmABCB3*^*+/−*^ parasites showed very little footpad inflammation, with minimal differences between infected and uninfected footpads.

### LmABCB3 is required for mitochondrial heme biosynthesis

**Fig. 6:**
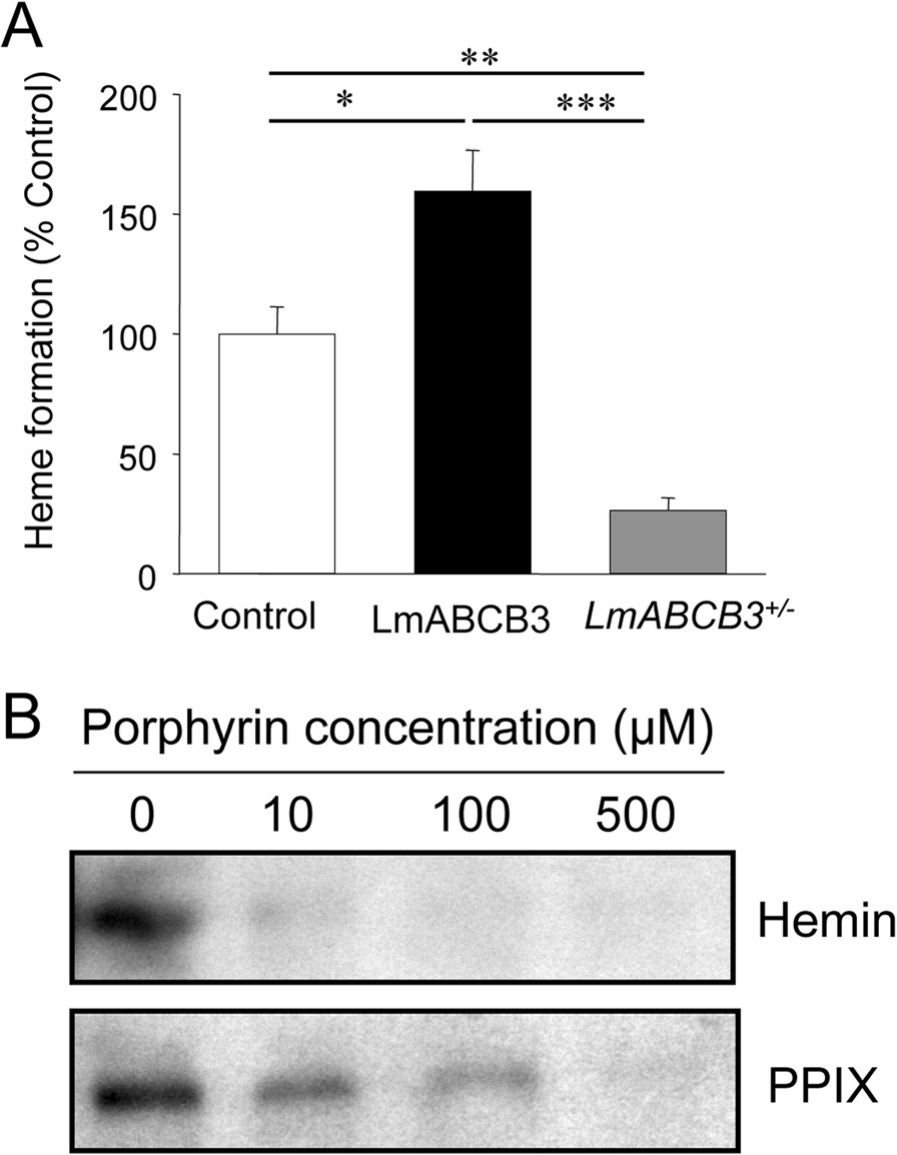
LmABCB3 is required for mitochondrial heme biosynthesis. **a** LmABCB3 cell levels correlate with the amount of heme synthesized from exogenous PPIX. The mitochondrial synthesis of heme from its precursor PPIX was measured as described in Experimental procedures after incubation of control, *LmABCB3* ^*+/−*^ and LmABCB3 overexpressing *L. major* promastigotes with 0.5 μM PPIX. The results represent the mean ± SEM of three independent experiments. * *p*<0.05, ** *p*<0.02, *** *p*<0.001. **b** LmABCB3 interacts with heme. Solubilized membrane proteins of *L. major* parasites overexpressing LmABCB3-GFP were subjected to a pull-down assay with hemin-agarose in the absence (0) or the presence of the indicated increasing concentration of free hemin (upper panel) or free PPIX (lower panel). LmABCB3-GFP was detected by inmmunoblotting with Anti-GFP antibody (1:5000)

We next analyzed whether the essential role of LmABCB3 in *L. major* was related to mitochondrial porphyrin trafficking, as shown for mammalian HsABCB6 [16]. Although *Leishmania* is auxotrophous for heme, it possesses a ferrochelatase gene (not present in *Trypanosoma*) [14] that adds iron to the precursor PPIX in the mitochondrial matrix to generate heme. This allows for an indirect quantification of the rate of mitochondrial porphyrin uptake via measurement of *de novo* heme synthesis from exogenous PPIX [7]. As shown in Fig. 6a, LmABCB3 overexpression increased the amount of heme synthetized from PPIX up to 60 %. In contrast, the deletion of one allele of *LmABCB3* diminished the amount of *de novo* heme formation by around 75 % with respect to control parasites.

We also explored the ability of LmABCB3 to bind heme using a pull-down assay, which demonstrated that detergent solubilized LmABCB3-GFP bound to hemin-agarose (Fig. 6b). This interaction was efficiently inhibited by 10 μM free hemin, whereas around 50-fold higher concentration of free PPIX was required to achieve similar inhibition of the LmABCB3-hemin agarose interaction (Fig. 6b). Altogether, these results provide clear evidence that LmABCB3 is involved in mitochondrial heme biosynthesis.

### LmABCB3 is required for the maturation of cytosolic iron/sulfur clusters

In addition to HsABCB6, LmABCB3 also showed similarity to HsABCB7. This protein and its orthologue in *S. cerevisiae*, ATM1, form part of the mitochondrial ISC export machinery and are essential in the generation of cytosolic ISC [22, 23]. Deletion of *ATM1* gene causes a drastic growth defect in yeast cells, including failure to grow on minimal medium and inability to use non-fermentable carbon sources [32, 60].

**Fig. 7:**
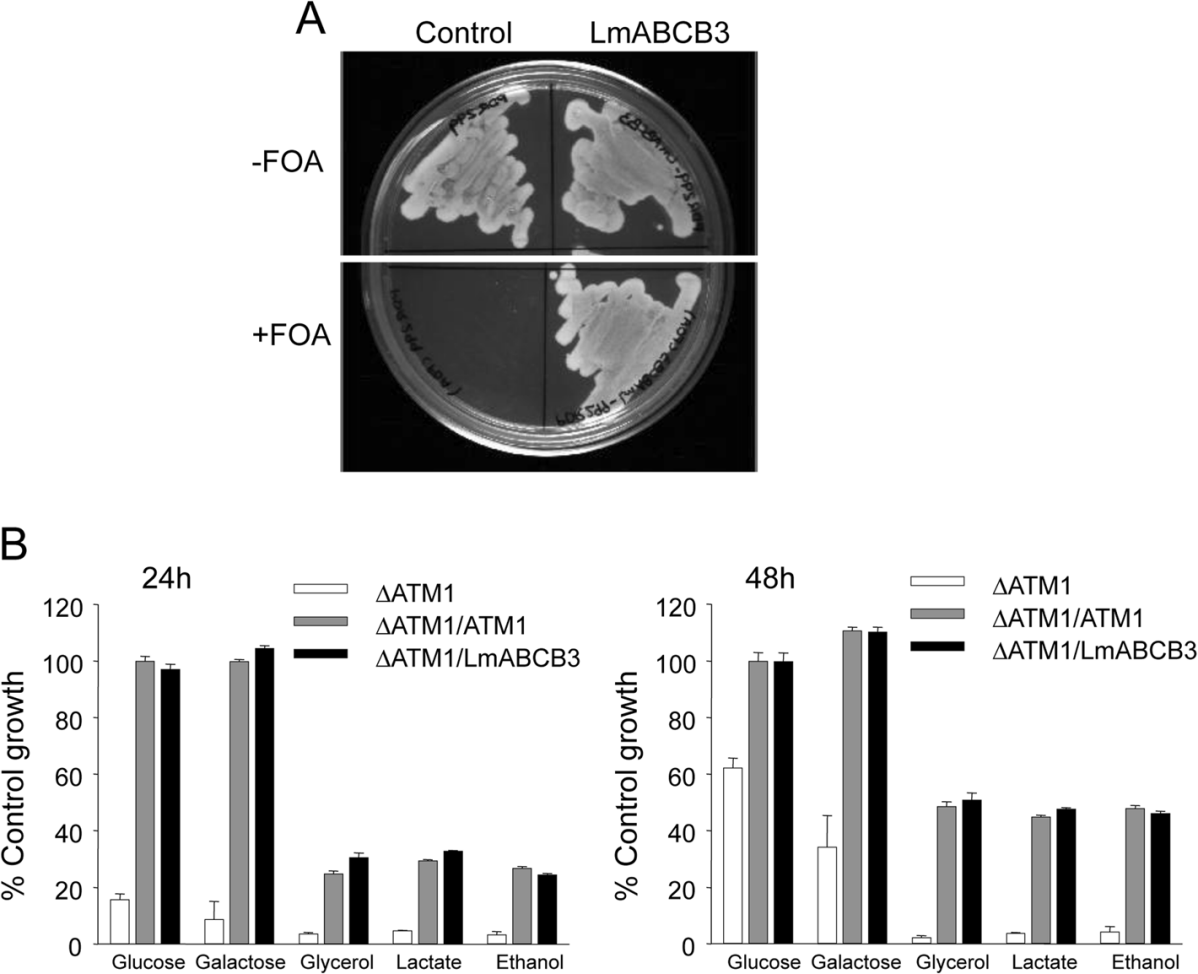
LmABCB3 completely rescue the severe growth defect phenotype of yeast lacking ScATM1. **a** LmABCB3 allows the normal growth of ΔATM1 yeast in minimal medium. ΔATM1/ATM1 (control) and (ΔATM1/ATM1 + LmABCB3 (LmABCB3) cells were plated on minimal (SD) glucose media containing (+FOA) or not (−FOA) 1 mg/ml 5′-fluoroorotic acid (FOA) and incubated at 30 °C for three days. **b** LmABCB3 allows ΔATM1 yeast to use of non-fermentable carbon sources. ΔATM1, ΔATM1/ATM1 and ΔATM1/LmABCB3 cells were diluted into rich (YP) media containing 2 % (w/v) glucose, 3 % (w/v) galactose, 2 % (v/v) glycerol, 2 % (w/v) lactate, or 2 % (v/v) ethanol to an A_600_ 0.05. After 24 (left) or 48 (right) hours of growth at 30 8C, the A_600_ was measured. Growth is expressed relative to ΔATM1/ATM1 growth in HC glucose

To analyze the ability of LmABCB3 to complement ScATM1 function in yeast we transfected Δ*ATM1* yeast containing a plasmid-encoded copy of *ScATM1*with a *URA3* marker (ΔATM1/ATM1) [36] with the yeast expression plasmid PDR299 containing either *LmABCB3* or no insert. Then, both yeast strains were plated in minimal medium with glucose containing or not 1 mg/ml 5′-fluoroorotic acid (FOA). This compound is converted to a toxic derivate in strains expressing the URA3 gene coding protein, allowing curing yeast strains of plasmid-encoded *ATM1* with *URA3* marker [36]. Figure 7a shows that ΔATM1 yeast cells transfected with PDR229 lacking LmABCB3 were unable to grow in FOA containing plates, whereas *ΔATM1* cells expressing LmABCB3 grew normally, indicating that LmABCB3 was able to functionally complement ScATM1. We also assessed the effect of LmABCB3 on the growth of *ΔATM1*cells in rich medium in the presence of different carbon sources. As expected [32, 36], *ΔATM1* yeast grew in fermentable (glucose and galactose) but not in non-fermentable (glycerol, lactate and ethanol) carbon sources, whereas *ΔATM1/ATM1* cells grew in all 5 (Fig. 7b). Interestingly, the expression of LmABCB3 was as effective as ScATM1 in restoring the growth of ΔATM1 yeast in both fermentable and non-fermentable carbon sources (Fig. 7b). These results strongly suggest that similar to ScATM1 and HsABCB7, LmABCB3 functions as a homodimer that localizes to the inner mitochondrial membrane with its NBD on the matrix side, and exports a substrate required for cytosolic ISC biogenesis.

Recently, the structure of ScATM1 [61] and a related bacterial transporter [62] have been solved. These proteins were crystalized in complex with GSH, in agreement with the proposed role of ScATM1 in the export of a GSH-ISC complex [29] or any other sulfur- and GSH- containing molecule to the cytosol [24]. GSH mainly interacts with ScATM1 by forming hydrogenen bonds with residues R^280^, R^284^ and D^398^ [61]. Interestingly, these residues are conserved in HsABCB7 (R^315^, R^319^ and E^433^) and LmABCB3 (R^479^, R^483^ and E^597^) (Additional file 5: Figure S3). Other ScATM1 residues surrounding bound GSH [61] were also generally conserved in the three transporters (Additional file 5: Figure S3). To further investigate this point, we have analyzed the predicted 3D structure of LmABCB3 by modeling methods.

**Fig. 8:**
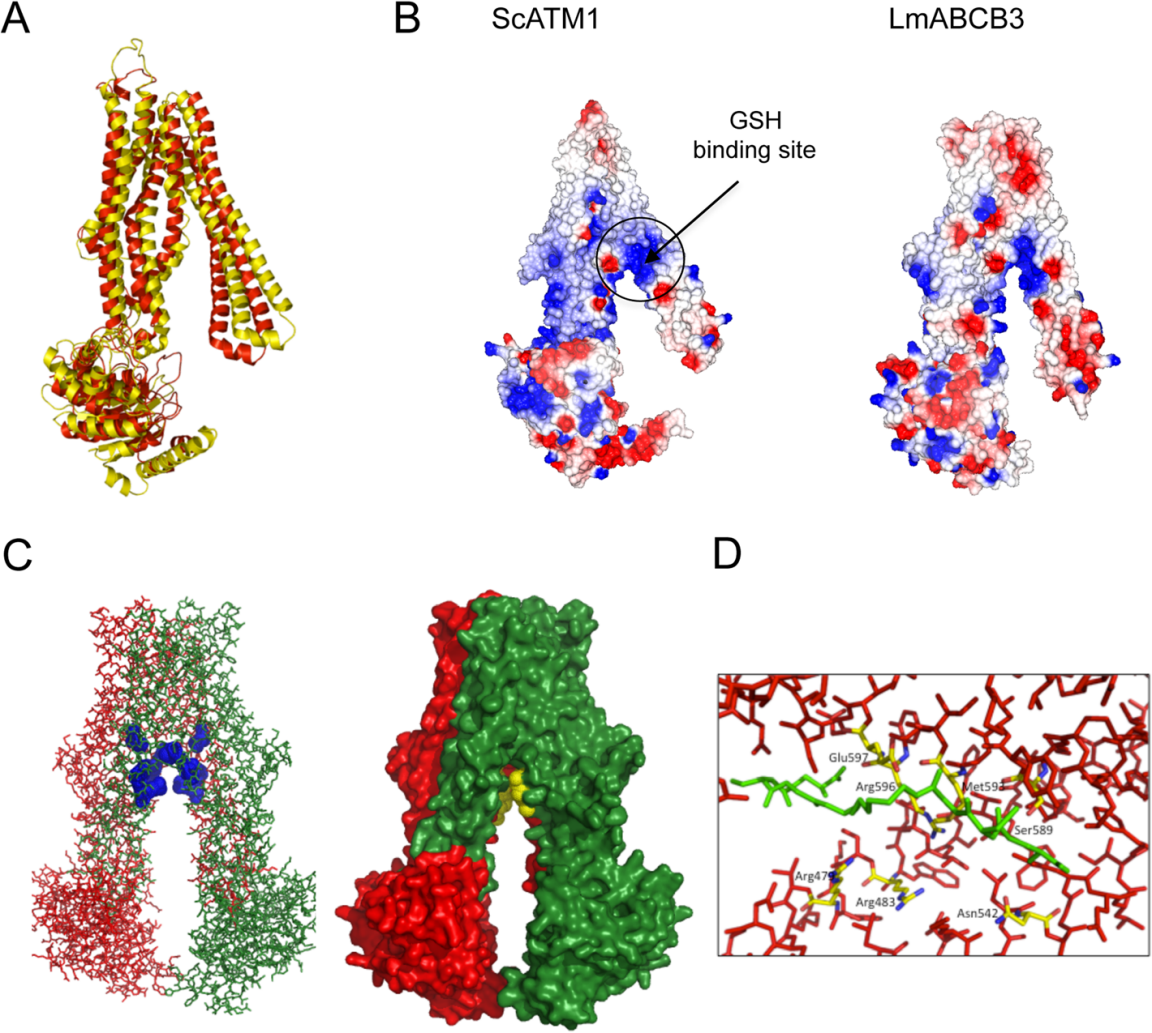
LmABCB3 homology model shares a thiol binding region with ScATM1 (**a**). Structural homology among LmABCB3 and ScATM1. A model for the 3D-structure of *L. major* ABCB3_∆UNE (aa 283–875) was built using Phyre2 molecular modelling server based on LmABCB3_∆UNE complete protein sequence, its predicted secondary structure and protein structures present in the PDB (Protein Data Bank) database. The structure of yeast ATM1 (pdb code: 1 MHY) was chosen by the server as the less divergent to the constructed LmABCB3 model with a 40 % sequence similarity. 3D-structural superimposition of yeast ATM1 structure (represented as yellow ribbons) and *L. major* ABCB3_∆UNE model (showed in red ribbons) was done using the DALI server (Z score: 26.5; RMSD:3.5 Å). **b** LmABCB3 conserves a positively charged pocket similar to the glutathione binding cavity present in ScATM1. Molecular surface representation colored by electrostatic charges (blue, positive; red, negative) distribution of yeast ATM1 and *L. major* ABCB3_∆UNEmodel. A positively charged pocket which binds a GSH molecule in ScATM1 is highlighted. An equivalent positive region is also observed in the proposed LmABCB3 model. **c** Trypanothione binding region in LmABCB3 dimer. Left. A model of LmABCB3_∆UNE dimer was built with chains A and B colored in red and green respectively. The residues conforming the T(SH)_2_ binding site of both monomers are highlighted in blue dots representation. Right. Molecular docking of reduced trypanothione (two molecules colored yelow) into the molecular surface of *L. major* ABCB3 dimer model (chains A and B colored red and green respectively) using Autodock4.0 program. **d** Close view of potential trypanothione binding site. The T(SH)_2_ molecule (colored green) appears surrounded by LmABCB3 conserved residues (yellow) in the conserved cavity of LmABCB3 model (monomer represented in red sticks)

Briefly, a model of LmABCB3_∆UNE (comprising aa 283–875, and lacking the UNE region) was built using Phyre2 molecular modeling server [45] based on protein structures present in the PDB (Protein Data Bank) database. The yeast ScATM1 was chosen by the server as having the structure closest to the LmABCB3_∆UNE model, with a 40 % sequence similarity. Next, we performed a 3D-structural superimposition of the yeast ScATM1 structure (PDB code: 1 MHY) and LmABCB3_∆UNE model using the DALI server [63] with good pairwise alignment parameters (Z score: 26.5; RMSD:3.5 Å) (Fig. 8a). In this configuration the GSH binding site in ScATM1 is located in a mainly positively charged pocket, which corresponds to a similar cavity in LmABCB3 with well-conserved residues disposed in equivalent positions (Fig. 8b). Since trypanosomatid parasites possess a unique redox metabolism which is mainly based on trypanothione (T(SH)_2_) [64], a molecule which contains two GSH, we hypothesized that LmABCB3 might be capable of binding T(SH)_2_. To evaluate this idea, docking calculations were carried out through Autodock 4.0 [65] using the LmABCB3_∆UNE 3D-model and reduced trypanothione as a potential ligand (Fig. 8c and d). Subsequent data analysis predicted that the T(SH)_2_ molecule could bind to the LmABCB3_∆UNE model with a favorable free energy change. Interestingly, the T(SH)_2_ binding region in LmABCB3 is located in close proximity to the equivalent charged pocket that binds GSH in yeast ATM1 (Fig. 8c), with the previously described conserved residues surrounding the T(SH)_2_ molecule (Fig. 8d). Significantly, a similar docking model built using the ScATM1 structural coordinates and GSH as a ligand predicted an interaction very similar to that found in the ScATM1 structure bound to GSH [61].

## Discussion

There is an urgent need to find new treatments against neglected tropical diseases. Over the past few years, the mitochondrion of protozoan parasites has emerged as a potent and promising goal for new therapeutic approaches (reviewed in [66, 67]). In addition to cellular respiration, this organelle plays essential roles related to regulation of cellular iron homeostasis, including the synthesis and trafficking of porphyrins and the generation of iron-sulfur clusters (ISC) [68, 69]. As iron is essential for trypanosomatids such as *Leishmania*, the route that controls its use is a potential target for drug development [70]. In mammals, two mitochondrial ABC half-transporters, ABCB6 and ABCB7, have been proposed to function in porphyrin transport and ISC assembly, respectively [20]. In this work we describe the functional characterization of a new *L. major* protein (LmABCB3), which shows significant sequence similarity with human ABCB6 and ABCB7.

Our results suggest that LmABCB3 likely functions in the maturation of cytosolic ISC, similar to human ABCB7, with which it shares 29.3 % sequence identity. Accordingly, LmABCB3 was able to completely rescue the severe growth defect of yeast lacking the HsABCB7 orthologue ATM1, including the growth in minimal medium and the ability to use non-fermentable carbon sources. These results also suggest that LmABCB3 works as a homodimer located in the inner mitochondrial membrane, with the NBD facing to the matrix, similar to ScATM1 and HsABCB7 [20, 32]. In mammals and yeast, it has been proposed that ABCB7/ATM1-like proteins transport a GSH-ISC complex [28, 29] or any other sulfur- and GSH- containing molecule [24] across the mitochondrial inner membrane that is then transferred to the cytosolic ICA machinery [28]. Indeed, structural studies with ScATM1 have shown that GSH binds to ScATM1 in a positively charged pocket [61], which corresponds to a similar cavity in the LmABCB3 model in which most of the residues involved in GSH binding are completely conserved. One of them, D^398^, which corresponds to E^597^ in LmABCB3, forms hydrogen bonds with GSH in ScATM1. Interestingly, mutation of the corresponding residue in HsABCB7 (E^433^) to lysine results in X-linked sideroblastic anemia and ataxia (XLSA/A) [23], highlighting the functional importance of the thiol interaction. Moreover, trypanosomatid parasites possess a unique redox mechanism based on the molecule trypanothione (T(SH)_2_) as a GSH substitute [64]. This parasite-specific dithiol is synthesized by the addition of two GSH molecules to spermidine and replaces the ubiquitous GSH system in the maintenance of redox homeostasis and xenobiotic detoxification [64]. Interestingly, T(SH)_2_ forms stable protein-free ISC species in vitro, suggesting a novel role of T(SH)_2_ as an intracellular ISC carrier [71]. In addition, docking analyzes indicate that T(SH)_2_ could interact with LmABCB3 similar to the way GSH does with ScATM1. This suggests that LmABCB3 could transport a T(SH)_2_-ISC complex or any other T(SH)_2_- containing molecule out of the mitochondrion for the maturation of cytosolic and nuclear iron-sulfur proteins, which further supports the proposed link between thiol-redox and iron metabolism in these organisms [71]. Determining whether this model is accurate or not will require elucidation of the structure of LmABCB3, but meanwhile it opens a new perspective in the understanding of LmABCB3 function.

In addition to its role in cytosolic ISC assembly, our data clearly describe an activity of LmABCB3 related to heme metabolism. We found that LmABCB3 can bind to heme, and that LmABCB3 protein levels correlated with the rate of mitochondrial heme production from cytosolic PPIX. This role is not shared by ScATM1 as both, synthesis and transport of heme, are functional in ΔATM1 yeast [32, 60]. LmABCB3 could therefore play a role in heme metabolism similar to human ABCB6, which is required for mitochondrial porphyrin import [16], and shares the highest level of sequence similarity (31 %) with LmABCB3 of any of the known mitochondrial ABC transporters. However, the ability of LmABCB3 to functionally complement a yeast *ATM1* deletion, indicating that it is likely oriented with its NBD on the matrix side of the mitochondrial inner membrane, argue against this possibility. This orientation predicts a probable export function, whereas ABCB6 is a porphyrin importer [16]. Alternatively, the effect of LmABCB3 on heme biosynthesis could reflect an essential interaction with ferrochelatase, as has been shown for the human ABCB7 [72]. Both in vitro and in vivo pull-down assays have demonstrated that ABCB7 interacts with the carboxy-terminus of ferrochelatase, and the transient expression of ABCB7 results in an increase in the enzymatic activity and the expression level of ferrochelatase, contributing to the production of heme during the differentiation of erythroid cells [72]. Finally, the results could be also explained if LmABCB3 exports *de novo* formed heme from the mitochondria, with less ability to transport PPIX, and increased levels of mitochondrial heme in LmABCB3^+/−^ cells inhibit ferrochelatase activity. Supporting this is the finding that around 50-fold more PPIX than hemin is required to compete with the interaction of LmABCB3 with hemin-agarose. In addition, at concentrations that may be physiologically relevant, heme has been shown to inhibit mammalian ferrochelatase in a non-competitive manner with respect to iron [73, 74]. Direct porphyrin transport assays with LmABCB3 reconstituted vesicles will be required to test this hypothesis. Other transporter such as RLIP76 mediates ATP-dependent transport of GSH conjugates as well as the hydrophobic compound doxorubicin [75], and even ABCG2, a mammalian ABC protein that export porphyrins [76], has been suggested to be also a GSH transporter [77].

Heme and ISC are co-factors required by a wide variety of essential enzymes involved in electron transport, enzyme catalysis and regulation of gene expression, and the ability to either synthesize or obtain them from the host is absolutely essential for the growth of trypanosomatid parasites [6, 14, 25, 78]. LmABCB3 has a role in both heme and ISC metabolism, and we provide several lines of evidence demonstrating these functions are essential for survival of trypanosomatid parasites. First, the inhibition of LmABCB3 via overexpression of a dominant negative allele is lethal for the parasite. Others and we have employed this strategy to study the function of an ABC half transporter taken advantage of its requirement of dimerization to become functional [7, 34, 59, 79]. However, to our knowledge, this is the first case of such a dramatic phenotype due to the dominant negative effect, indicating that the low level of functional Wt/Wt LmABCB3 dimers expected using this strategy is not sufficient to allow parasite growth. Second, although heterozygous deletion of one *lmABCB3* allele slightly alters promastigotes growth, it prevents *L. major* replication within macrophages. A double KO line could not be obtained, presumably due to its lethal effect. Consistent with an essential role of LmABCB3 in intracellular amastigotes, the elimination of one *LmABCB3* allele severely reduce parasite virulence in a mousemodel of cutaneous leishmaniasis. Thus, even though low LmABCB3 levels support the growth of axenic *LmABCB3*^*+/−*^ promastigotes, they are not sufficient for intracellular amastigotes to cause disease in mice. Although the involvement of *Leishmania* ISC- and/or heme-containing proteins in parasite virulence is not explored, this differential effect on both parasite stages could be explained by a more relevant role of the machinery involved in cytosolic ISC and/or heme metabolism in the amastigote stage. In fact, axenic promastigotes do not require to produce heme as it is taken from the culture medium [6], whereas heme synthesis from macrophage coproporphyrinogen has been proposed to be functional in intracellular amastigotes [14, 15]. In contrast, results from high-throughput RNAi target sequencing in *T. brucei* [80] suggest that down regulation of TbABCB3 does not affect cell proliferation of neither bloodstream nor procyclic trypomastigote stages. These results need to be confirmed by a specific RNAi assay, but they must been seen as quite unexpected in the case LmABCB3 and TbABCB3 were functional orthologues. Although *Trypanosoma* does not synthetize heme from precursors, it requires cytosolic ISC to survive [27, 81]. 

Finally, a detailed analysis of the sequence of the transcribed LmABCB3 mRNA [44] predicts an unusual topology that includes a UNE region not found in any other protein outside the genus *Leishmania*. This unique extension includes a consensus MLRR motif followed by a hydrophobic region rich in Ala, Leu, and Val, which has been proposed to allow for recognition and import via the mitochondrial import apparatus of trypanosomatid parasites [56]. Our results have confirmed that the UNE region is required for the proper localization of LmABCB3 to mitochondria. Interestingly, the UNE is also predicted to contain a conserved metal-binding domain (TRASH), with a well-conserved cysteine motif probably involved in metal coordination [50]. TRASH domains are known to function in metal sensing, trafficking, and heavy-metal resistance [50]. The precise role of this domain in LmABCB3 remains to be elucidated, but based on its association with proteins involved in metal homeostasis, it is likely to be important for the function of LmABCB3 in heme/ISC metabolism.

## Conclusion

We have shown that the novel *Leishmania* mitochondrial transporter LmABCB3 is the first ABC transporter described to be essential in any trypanosomatid parasite. We have provided solid evidences suggesting strongly that LmABCB3 plays critical roles in both mitochondrial heme and cytosolic ISC biogenesis that explain its essentiality. Based on the demonstration that diminution in functional LmABCB3 levels is deleterious for the parasite, agents that specifically inactivate LmABCB3 would likely be lethal for these pathogens, even in the absence of complete inhibition. The unique nature of the 20 kDa N-terminal peptide, including its requirement for mitochondrial import and the presence of a TRASH domain, makes it an outstanding candidate for the use of rational design methods for the development of specific LmABCB3 inhibitors to be used against *Leishmania,* responsible of a neglected disease that affects some of the poorest people on the planet.

### Note added in proof

While our manuscript was under revision, Horáková and coworkers reported the characterization of TbAtm, the putative *T. brucei* ortholog of LmABCB3 [82]. The authors showed that TbAtm is involved in cytosolic Fe-S cluster assembly but not in mitochondrial heme metabolism in procyclic forms of *T. brucei*. Contrary to our observations following the depletion of LmABCB3 in *Leishmania*, depletion of TbAtm led to limited growth defects in procyclic parasites.

## Additional files

Additional file 1: Table S2. Primer used in this study. Restriction enzyme sites are in bold and underlined. (TIF 657 kb) Additional file 2: Figure S1. The UNE domain is exclusive of *Leishmania* ABCB3. Schematic representation of putative mitochondrial ABCB transporters showing the unique N-terminal extension (UNE), the Transmembrane Domain (TMD), the Nucleotide Binding Domain (NBD) and the theoretical molecular weight. Lm: *L. major*; Li: *L. infantum*; Lmx: *L. mexicana*; Lb: *L. braziliensis*; Tb: *T. brucei*; Tc: *T. cruzi*; Hs: *H. sapiens*; Sc: *S. cereviciae*. (TIF 1523 kb) Additional file 3: Table S1. Homology between different putative mitochondrial ABCB half-trransporters. Analysis was performed with Clustal W (http://www.ncbi.nlm.nih.gov/protein/). Analyzed proteins (GenPept accession number in brackets) were from *Leishmania major*: LmABCB3 (XP_001685635,1), LmABCB1 (XP_001683806.1); *Trypanosoma brucei*: TbABCB3 (XP_829749.1), TbABCB1 (XP_828146,1); *Trypanosoma cruzi*: TcABCB3 (XP_811319.1), TcABCB1(XP_820554.1); *Homo sapiens*: HsABCB6 (NP_005680), HsABCB7 (NP_001258628.1), HsABCB8 (NP_001269222.1), HsABCB10 (NP_036221) and *Saccharomyces cerevisiae*: ScATM1 (NP_014030.1), ScMDL1 (NP_013289.1) and ScMDL2 (NP_015053.2). Data indicate the percentage of identity (grey) and similarity (white) between protein sequences. (TIF 1290 kb) Additional file 4: Figure S2. Expression of LmABCB3-GFP and LmABCB3_∆UNE-GFP. Western blot analysis of total protein from LmABCB3-GFP (lane 1) or LmABCB3_∆UNE-GFP (lane 2) and GFP (lane 3) expressing *L. major* parasites. Immunodetection were performed with antibody anti-GFP incubation at a 1:5000 dilution. The molecular mass standards (kDa) from Bio-Rad are indicated on the left. (TIF 2482 kb) Additional file 5: Figure S3. The ScATM1 residues that interact with glutathione are conserved in LmABCB3. The alignment of the indicated amino acids of *S. cerevisiae* ScATM1, *H. sapiens* HsABCB7 and *L. major* LmABCB3 (ClustalW software) shows that LmABCB3 share the ScATM1 residues forming hydrogenen bonds with GSH (highlighted in yellow). The E433 residue of HsABCB7 mutated to lysine in XLSA/A patients is indicated by a red arrow. Other ScATM1 residues surrounding bound GSH are highlighted in green. Identical (*), strongly similar (:) and weakly similar (.) amino acids are coloured in red, green and blue, respectively. (TIF 379 kb)
