# Supplementary figures and images for: LmABCB3, an atypical mitochondrial ABC transporter essential for Leishmania major virulence, acts in heme and cytosolic iron/sulfur clusters biogenesis
Source: Parasit Vectors. 2016 Jan 5;9:7. doi: 10.1186/s13071-015-1284-5 (PMC4700571; doi:10.1186/s13071-015-1284-5)

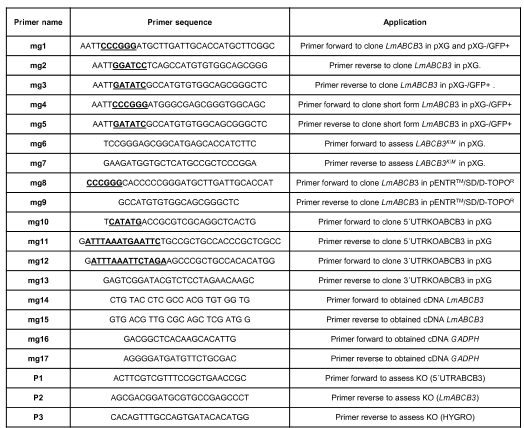

Supplement: Additional file 1: Table S2. — Primer used in this study. Restriction enzyme sites are in bold and underlined. (TIF 657 kb) [file 13071_2015_1284_MOESM1_ESM.tif]

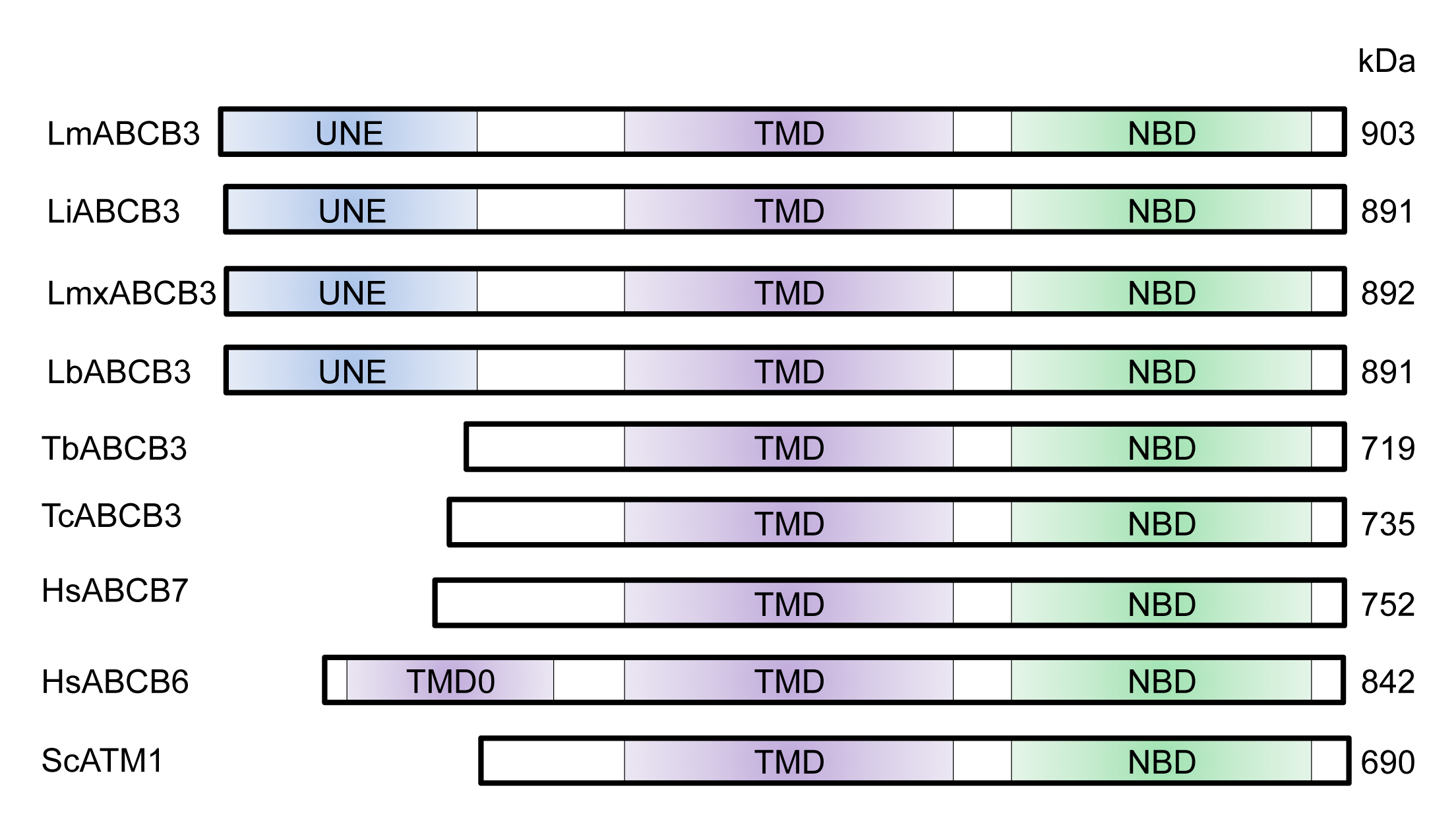

Supplement: Additional file 2: Figure S1. — The UNE domain is exclusive of Leishmania ABCB3. Schematic representation of putative mitochondrial ABCB transporters showing the unique N-terminal extension (UNE), the Transmembrane Domain (TMD), the Nucleotide Binding Domain (NBD) and the theoretical molecular weight. Lm: L. major; Li: L. infantum; Lmx: L. mexicana; Lb: L. braziliensis; Tb: T. brucei; Tc: T. cruzi; Hs: H. sapiens; Sc: S. cereviciae. (TIF 1523 kb) [file 13071_2015_1284_MOESM2_ESM.tif]

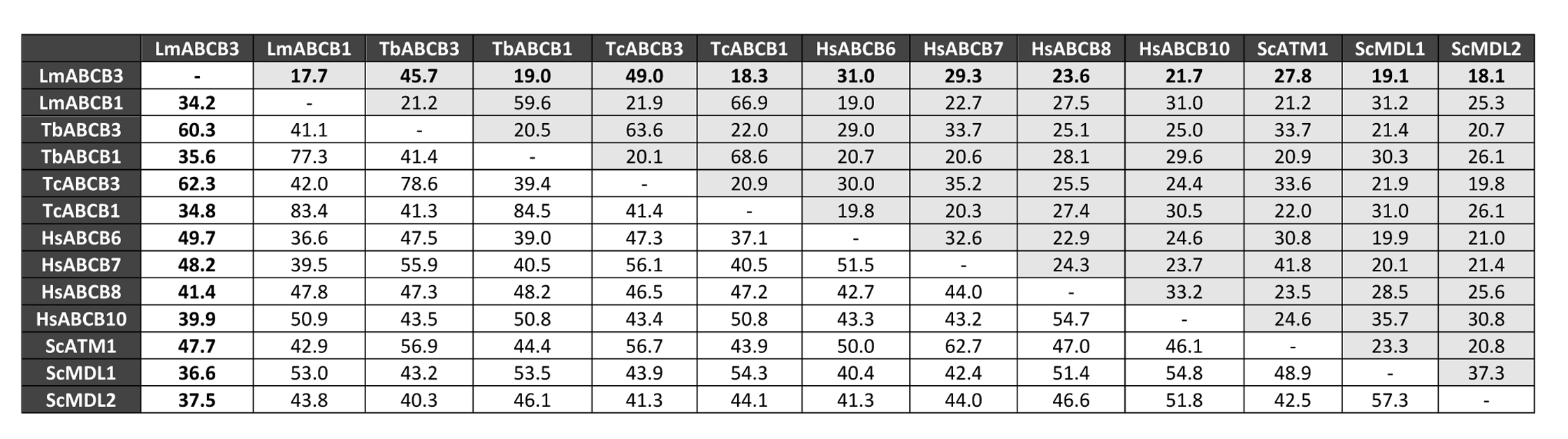

Supplement: Additional file 3: Table S1. — Homology between different putative mitochondrial ABCB half-trransporters. Analysis was performed with Clustal W (http://www.ncbi.nlm.nih.gov/protein/). Analyzed proteins (GenPept accession number in brackets) were from Leishmania major: LmABCB3 (XP_001685635,1), LmABCB1 (XP_001683806.1); Trypanosoma brucei: TbABCB3 (XP_829749.1), TbABCB1 (XP_828146,1); Trypanosoma cruzi: TcABCB3 (XP_811319.1), TcABCB1(XP_820554.1); Homo sapiens: HsABCB6 (NP_005680), HsABCB7 (NP_001258628.1), HsABCB8 (NP_001269222.1), HsABCB10 (NP_036221) and Saccharomyces cerevisiae: ScATM1 (NP_014030.1), ScMDL1 (NP_013289.1) and ScMDL2 (NP_015053.2). Data indicate the percentage of identity (grey) and similarity (white) between protein sequences. (TIF 1290 kb) [file 13071_2015_1284_MOESM3_ESM.tif]

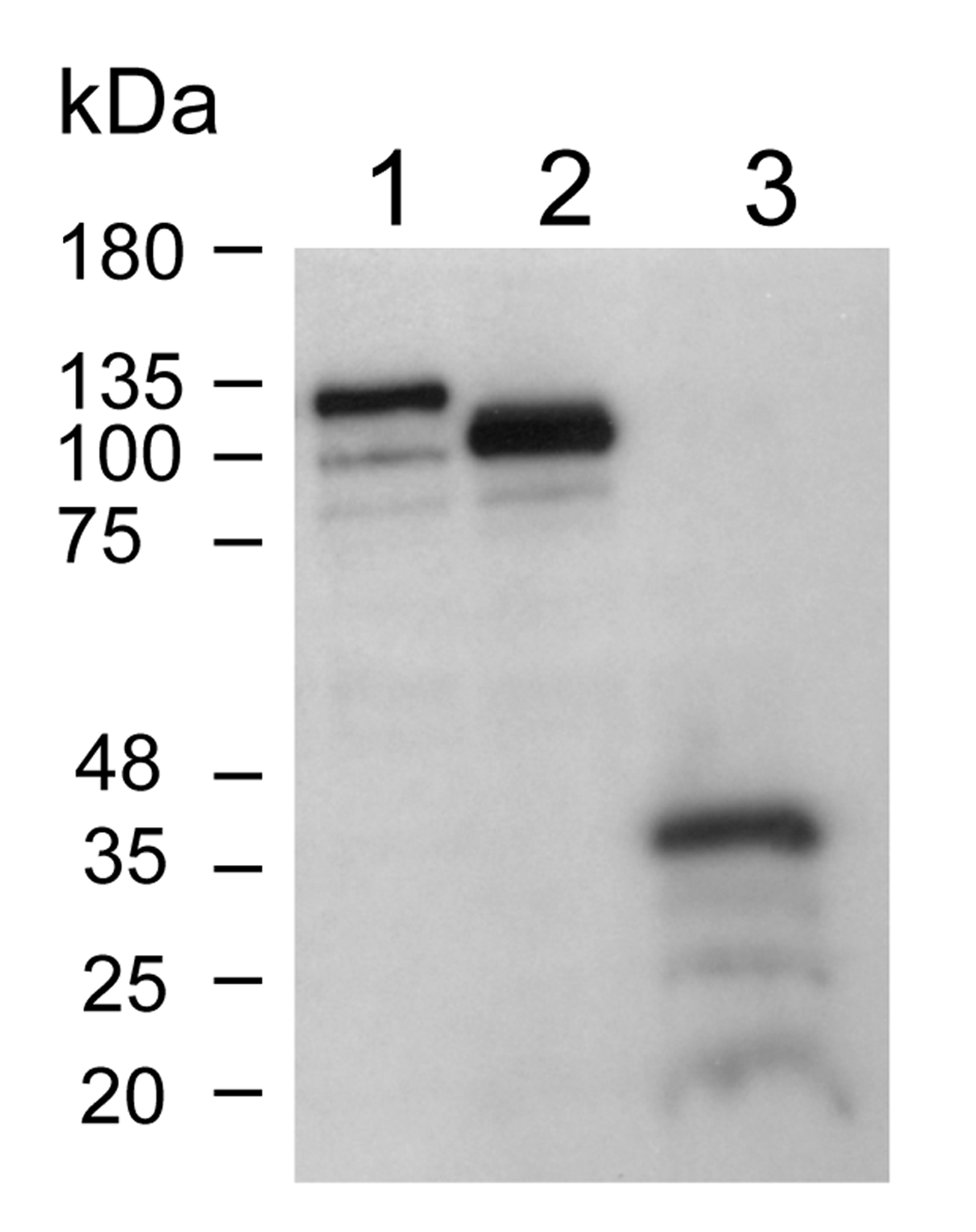

Supplement: Additional file 4: Figure S2. — Expression of LmABCB3-GFP and LmABCB3_∆UNE-GFP. Western blot analysis of total protein from LmABCB3-GFP (lane 1) or LmABCB3_∆UNE-GFP (lane 2) and GFP (lane 3) expressing L. major parasites. Immunodetection were performed with antibody anti-GFP incubation at a 1:5000 dilution. The molecular mass standards (kDa) from Bio-Rad are indicated on the left. (TIF 2482 kb) [file 13071_2015_1284_MOESM4_ESM.tif]

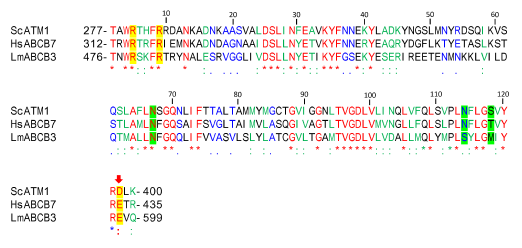

Supplement: Additional file 5: Figure S3. — The ScATM1 residues that interact with glutathione are conserved in LmABCB3. The alignment of the indicated amino acids of S. cerevisiae ScATM1, H. sapiens HsABCB7 and L. major LmABCB3 (ClustalW software) shows that LmABCB3 share the ScATM1 residues forming hydrogenen bonds with GSH (highlighted in yellow). The E433 residue of HsABCB7 mutated to lysine in XLSA/A patients is indicated by a red arrow. Other ScATM1 residues surrounding bound GSH are highlighted in green. Identical (*), strongly similar (:) and weakly similar (.) amino acids are coloured in red, green and blue, respectively. (TIF 379 kb) [file 13071_2015_1284_MOESM5_ESM.tif]
